# 

**Published:** 1892-03

**Authors:** 


### CONTENTS.

### COMMUNICATIONS.

Combination Fillings........ 99
Erosion ........ 108
Ichoræmia........ 116
Prophylaxis........ 118
The Use of Antiseptics for Sterilizing Cavities before Filling ........ 127
Some Practical Points in Regard to Methods in Construction of Crown and Bridge Work........ 131
The Treatment of Teeth During Pregnancy........ 138

### PROCEEDINGS.

Walnut Hills Medical Society........ 144
Antisepsis for the Hands........ 145
A Higher Professional Standard........ 146

### EDITORIAL.

The World's Columbian Exposition........ 148
Bibliographical........ 150
Professional—Change of Location........ 150

